# Growth Inhibition of Human Gynecologic and Colon Cancer Cells by *Phyllanthus watsonii* through Apoptosis Induction

**DOI:** 10.1371/journal.pone.0034793

**Published:** 2012-04-20

**Authors:** Sujatha Ramasamy, Norhanom Abdul Wahab, Nurhayati Zainal Abidin, Sugumaran Manickam, Zubaidah Zakaria

**Affiliations:** 1 Institute of Biological Sciences, Faculty of Science, University of Malaya, Kuala Lumpur, Malaysia; 2 Centre for Foundation Studies in Science, University of Malaya, Kuala Lumpur, Malaysia; 3 Hematology Unit, Cancer Research Centre, Institute for Medical Research, Kuala Lumpur, Malaysia; Wayne State University School of Medicine, United States of America

## Abstract

*Phyllanthus watsonii* Airy Shaw is an endemic plant found in Peninsular Malaysia. Although there are numerous reports on the anti cancer properties of other *Phyllanthus* species, published information on the cytotoxicity of *P. watsonii* are very limited. The present study was carried out with bioassay-guided fractionation approach to evaluate the cytotoxicity and apoptosis induction capability of the *P. watsonii* extracts and fractions on human gynecologic (SKOV-3 and Ca Ski) and colon (HT-29) cancer cells. *P. watsonii* extracts exhibited strong cytotoxicity on all the cancer cells studied with IC_50_ values of ≤ 20.0 µg/mL. Hexane extract of *P. watsonii* was further subjected to bioassay-guided fractionation and yielded 10 fractions (PW-1→PW-10). PW-4→PW-8 portrayed stronger cytotoxic activity and was further subjected to bioassay-guided fractionation and resulted with 8 sub-fractions (PPWH-1→PPWH-8). PPWH-7 possessed greatest cytotoxicity (IC_50_ values ranged from 0.66 – 0.83 µg/mL) and was selective on the cancer cells studied. LC-MS/MS analysis of PPWH-7 revealed the presence of ellagic acid, geranic acid, glochidone, betulin, phyllanthin and sterol glucoside. Marked morphological changes, ladder-like appearance of DNA and increment in caspase-3 activity indicating apoptosis were clearly observed in both human gynecologic and colon cancer cells treated with *P. watsonii* especially with PPWH-7. The study also indicated that *P. watsonii* extracts arrested cell cycle at different growth phases in SKOV-3, Ca Ski and HT-29 cells. Cytotoxic and apoptotic potential of the endemic *P. watsonii* was investigated for the first time by bioassay-guided approach. These results demonstrated that *P. watsonii* selectively inhibits the growth of SKOV-3, Ca Ski and HT-29 cells through apoptosis induction and cell cycle modulation. Hence, *P. watsonii* has the potential to be further exploited for the discovery and development of new anti cancer drugs.

## Introduction

There is a long history in the usage of plants in Southeast Asian countries, some of which have been proven to possess interesting biological activities with potential therapeutic applications [1]. The use of plants as medicine has resulted in the isolation and characterization of pharmacologically active compounds [2] and today there are at least 120 distinct chemical substances derived from plants that are considered as important drugs and active ingredients in the pharmaceutical industry [3].

Cancer is one of the leading causes of death in both developed and developing countries and continues to be a major public health problem in many parts of the world [4]. Much effort has been made to develop various approaches to reduce the threat caused by cancer and only modest progress has been made in reducing the morbidity and mortality of this dreadful disease [5]. According to the world cancer report released by the International Agency for Research on Cancer, globally there were 12.4 million new cancer cases in 2008 (6,672,000 in men and 5,779,000 in women) and 7.6 million deaths from cancer (4,293,000 in men and 3,300,000 in women) [6]. At present, cancer treatment by chemotherapeutic agents, surgery and radiation have not been fully effective against the high incidence or low survival rate of most cancers [7]. The search for new anti cancer agents from plant sources is one of the realistic and promising approaches in the field of cancer chemoprevention and this led to the discovery of many novel anti cancer drugs, including vinca alkaloids, vinblastine and vincristine, taxol, camptothecins, and podophyllotoxins [8]. Approaches have also been taken towards discovering anti cancer agents from tropical plants [2] as alternative cancer remedies because of their low toxicities and costs [9].

During the last few decades, many studies have indicated that the mechanism of action of many anti cancer drugs is based on apoptosis induction, and thus opening a new strategy in the search of anti cancer drugs [10]. An apoptotic induction is a highly desirable characteristic of treatment strategy for cancer control, it is therefore important to screen apoptotic inducers from plants, either in the form of crude extracts or as component compounds [11]. These kinds of studies must be done by evaluating the cytotoxicity and apoptotic induction in cancer cell lines before whole animal studies or clinical trials were carried out.

**Figure 1:**
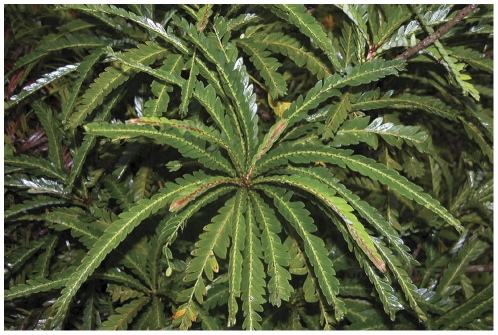
*Phyllanthus watsonii*.

*Phyllanthus watsonii* Airy Shaw is a small shrub growing to about 1 m high and belongs to the family Phyllanthaceae (Figure 1). The species is a narrow endemic and is only known to occur from northern Johor to southern Pahang (two states in Peninsular Malaysia) on the banks of Endau River [1], [12]. A number of studies have shown that extracts and compounds derived from other *Phyllanthus* species could suppress the growth of various cancers including cervical [13]; liver [14], lung [15], macrophages [16], uterine, gastric [17], breast and colon cancer [18]. However, there were no detailed study been carried out to evaluate the cytotoxic and apoptotic effects of *P. watsonii* on human cancer cell lines, except one report demonstrating the effect of the aqueous and methanol extract of *P. watsonii* against MeWo skin melanoma cells and PC-3 prostate cancer cells [19]. Phytochemistry studies revealed the presence of two new unsaturated nor-triterpenes (26-nor-D:A-friedoolean-14-en-3-one and 26-nor-D:A-friedoolean-14-en-3beta-ol), lupenyl palmitate, friedelin, epifriedelanol, glochidone, glochidonol, lupeol, lup-20(29)-en-1 beta, 3 beta-diol, sitosterol and sitosterol-beta-(D)-glucoside in *P. watsonii* [20].

The present study was carried out to evaluate the cytotoxic potential of methanol, hexane and ethyl acetate extracts of *P. watsonii* against human gynecologic and colon cancer cell lines. MRC-5, a normal human lung fibroblast cell line was also used to determine the specificity of the extracts for cancerous cells. MRC-5 cells have been commonly used as a control in many similar studies [21]–[23]. Bioassay-guided fractionation was also carried out in order to determine the bioactive secondary metabolites with cytotoxic properties. To our knowledge, this will be the first time such extracts and fractions from *P. watsonii* are being tested against these cell lines. In addition the apoptotic process associated with the cytotoxic effect in these cells was also investigated.

## Materials and Methods

### Ethics Statement

All necessary permits and permission for collection of materials were obtained for the described field studies and the party involved is duly acknowledged. The species (*Phyllanthus watsonii*) is located in the Endau Rompin National Park which belongs to the state government of Johor and managed by the Johor National Parks Corporation (JNPC), Malaysia. The species is not endangered and the habitat is not threatened and it is also not listed in the appendices of CITES (Convention on International Trade in Endangered Species of Wild Fauna and Flora).

### Plant Materials

The leaves of *Phyllanthus watsonii* were collected from Endau Rompin National Park, Johor (Peninsular Malaysia) in June 2008. Authentication of *P. watsonii* was carried out in the herbarium of the Rimba Ilmu Botanical Garden, Institute of Biological Sciences, University of Malaya and a voucher material (Ref. No. KLU46068) for this study was deposited at the same herbarium.

### Preparation of Extracts from *P. watsonii*

The dried leaves of *P. watsonii* (PW) (2 kg) were ground and extracted with methanol (MeOH) (Fisher Scientific, UK) (6 L × 3 times) at room temperature for 72 hours and filtered through Whatman No. 1 filter paper (Whatman, England). The MeOH filtrate was collected and excess solvent was evaporated under reduced pressure using a rotary evaporator (Buchi, Switzerland) at 40°C to dryness producing 160.3 g of dark-greenish MeOH extract (PW-M). Part of the MeOH extract was reserved for the assay while the remaining portion was further shaken vigorously with hexane (Fisher Scientific, UK) until the resultant hexane became almost colorless. The hexane soluble solution was filtered and pooled, followed by concentration under reduced pressure by a rotary evaporator to yield 6.5 g of hexane extract (PW-H). The remaining hexane insoluble was subjected to solvent-solvent extraction with a mixture of ethyl acetate (EtOAc) (Fisher Scientific, UK) and distilled water (1∶1, v/v) followed by fairly vigorous mixing. This mixture was then successively fractionated using a separating funnel in which two distinct layers formed. The bottom layer (water layer) was discarded while the EtOAc phase (top layer) was released into a clean beaker. This filtrate was concentrated under reduced pressure using a rotary evaporator to yield 9.7 g of EtOAc extract (PW-E). In all experiments, extracts were dissolved in dimethylsulfoxide (DMSO) (Sigma) as stock solution and stored at −20°C. Prior to analysis, the final concentration of each sample was prepared by diluting the stock solution in 10% DMSO. The final concentration of DMSO in the cell culture was <0.5%. PW-M, PW-H and PW-E of *P. watsonii* were further subjected to Neutral Red Uptake assay according to the procedures described later. PW-H was found to exhibit strongest cytotoxicity when compared with PW-M and PW-E and warranted further investigation.

### Bioassay-guided Fractionation of PW-H

**Figure 2:**
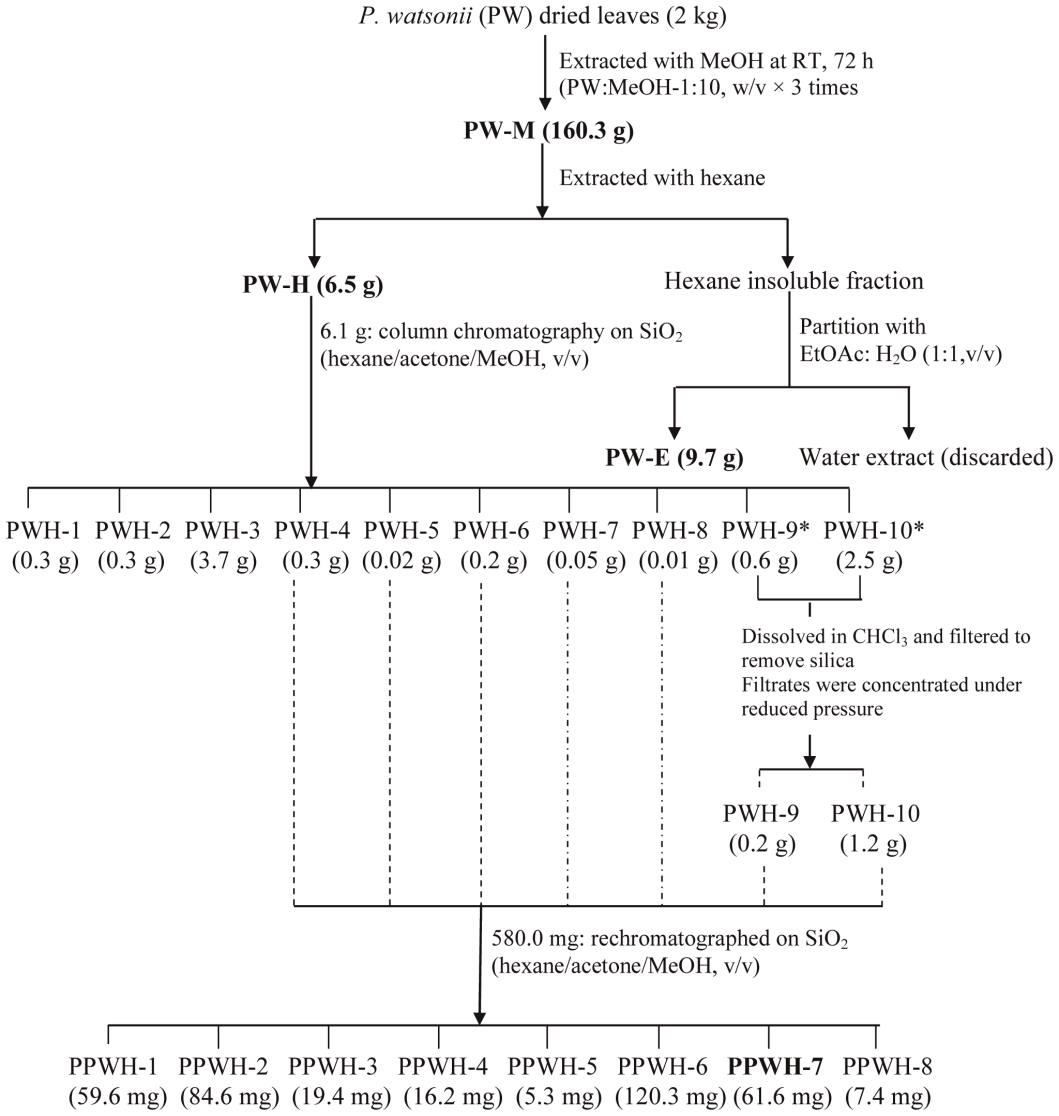
Flow chart of extraction procedure for *P. watsonii*.

The cytotoxically active fraction of PW-H (6.1 g) was chromatographed on a column (4 cm i.d. × 40 cm length) packed with silica gel 60 F254, 0.25 mm thickness (Merck, Darmstadt, Germany) (160 g). Gradient step elution began with 100% hexane and polarity of eluting solvent was gradually increased using acetone (Me_2_CO) (Fisher Scientific, UK) and MeOH. Eluents of 25 mL volume were collected in numbered vials. The separation of each eluent was monitored on pre-coated thin layer chromatography (TLC) silica gel 60 F254 plate using n-hexane/acetone (20∶80, v/v) as a developing solvent and the TLC zones were detected after spraying with *p*-anisaldehyde reagent and heating at 105°C for 5 – 10 min. The eluents were pooled according to the similarity of the chemical composition detected on TLC and the excess solvent was evaporated under reduced pressure using a rotary evaporator to yield a total of 10 fractions designated as PWH-1, PWH-2, PWH-3,…….,PWH-8, PWH-9* and PWH-10* (Figure 2). Fractions PWH-9* and PWH-10* were found to contain silica gel and were further dissolved in chloroform and filtered through Whatman No. 1 filter paper (Whatman, England) to remove the silica. Fractions PWH-9 and PWH-10 were obtained after removing the excess solvent by evaporation to dryness at 40°C under reduced pressure in a rotary evaporator. All the fractions were further subjected to Neutral Red Uptake assay (procedures described later) and fractions PWH-4,…..,PWH-8 revealed as the most active fractions in cytotoxic activity.

These fractions (PWH-4,…..,PWH-8) (581.0 mg) were pooled and further purified with silica column chromatography (2.6 cm i.d. × 60 cm length) packed with silica gel 60 F254, 0.25 mm thickness (Merck, Darmstadt, Germany) (160 g). Elution began with 100% hexane and polarity of eluting solvent was gradually increased using Me_2_CO and MeOH. Eluents of 25 ml volume were collected in numbered vials. The separation of each eluent was monitored on pre-coated thin layer chromatography (TLC) silica gel 60 F254 plate using n-hexane/acetone (20∶80, v/v) as developing solvent and the TLC zones were detected after spraying with *p*-anisaldehyde reagent and heating at 105°C for 5 – 10 min. The eluents were pooled according to the similarity of the chemical composition detected on TLC and the excess solvent was evaporated under reduced pressure using a rotary evaporator to yield a total of 8 sub-fractions designated as PPWH-1, PPWH-2,…..,PPWH-8. The sub-fractions were subjected to Neutral Red Uptake assay as described later. The solvent system profile and the yield of each fractions and sub-fractions obtained are summarized in Table 1.

**Table 1:** Solvent system profile and yield (%) of obtained fractions and sub-fractions.

Fractions/Sub-fractions	Solvent system for CC	Ratio(v/v)	Yield(%)
aPWH-1	hexane 100%	100	5.49
PWH-2	hexane/Me_2_CO	40 ∶ 60	4.67
PWH-3	hexane/Me_2_CO	40 ∶ 60	60.64
PWH-4	hexane/Me_2_CO	40 ∶ 60	5.13
PWH-5	hexane/Me_2_CO	40 ∶ 60	0.30
PWH-6	hexane/Me_2_CO	40 ∶ 60	3.10
PWH-7	hexane/Me_2_CO	40 ∶ 60	0.83
PWH-8	hexane/Me_2_CO; hexane/ Me_2_CO	40 ∶ 60; 50 ∶ 50	0.16
PWH-9	hexane/Me_2_CO; Me_2_CO/MeOH	50 ∶ 50; 40 ∶ 60	8.83
PWH-10	Me_2_CO/MeOH; Me_2_CO/MeOH	40 ∶ 60; 90 ∶ 10	41.71
bPPWH-1	hexane 100%	100	10.26
PPWH-2	hexane/Me_2_CO	90 ∶ 10	14.56
PPWH-3	hexane/Me_2_CO	90 ∶ 10	3.34
PPWH-4	hexane/Me_2_CO	90 ∶ 10	2.79
PPWH-5	hexane/Me_2_CO; hexane/ Me_2_CO	90 ∶ 10; 60 ∶ 40	0.91
PPWH-6	hexane/Me_2_CO	60 ∶ 40	20.71
PPWH-7	hexane/Me_2_CO	50 ∶ 50	10.61
PPWH-8	hexane/Me_2_CO; hexane/ Me_2_CO	50 ∶ 50; 40 ∶ 60	1.27

a PWH-1, PWH-2,…….,PWH-10: fractions of PW-H of *P. watsonii.*

b PPWH-1, PPWH-2,….,PPWH-8: sub-fractions from pooled fractions (PWH-4,…,PWH-8).

Subsequently, cytotoxically active extracts (PW-M, PW-H and PW-E) and most active sub-fraction (PPWH-7) were selected and further evaluated for its apoptotic effect towards the gynecologic and colon cancer cell lines.

### LCMS-MS Analysis

The most active sub-fraction, PPWH-7 was analyzed by LC-MS/MS system equipped with the 3200 QTrap mass spectrometer (Applied Biosystem, Darmstadt, Germany) and Shidmazu UHPLC system. The chromatographic separation was performed on a 50 mm × 2.0 mm × 5 µM Aqua C18 column (Phenomenex, Torrance, CA), eluted with a mobile phase consisting of water (A) and acetonitrile (B) containing 0.2% formic acid and 2 mM ammonium formate. A gradient elution (starting from 10% of A to 90% of B, from 0.01 min to 10.0 min, hold for 2 min and back to 10% of A in 0.1 min and re-equilibrated for 5 min) was used to separate the compounds of interest prior to mass spectral analysis. The mass spectrometer analysis was performed in a positive ion mode (*m/z* M + H^+^) for detection of secondary compounds. Identities of the compounds were obtained by matching their molecular ions (*m/z*) obtained by LC-MS/MS with reference standards where available and by correlation with previous published data on chemical constituents from *Phyllanthus* [24]–[30].

### Cell Culture

The human cell lines SKOV-3 (ovarian carcinoma cell line), Ca Ski (epidermal carcinoma of cervix cell line), HT-29 (colon cancer cell line) and MRC-5 (normal lung fibroblast cell line) were purchased from American Type Culture Collection (ATCC), USA. SKOV-3 cells were cultured in Dulbecco’s Modified Eagle’s Medium (DMEM) (Sigma, UK), whereas HT-29, Ca Ski and MRC-5 cells were cultured in RPMI 1640 medium (Sigma, UK) and DMEM medium (Sigma, UK), respectively. All the media were supplemented with 10% (v/v) fetal bovine serum (FBS) (PAA Lab., Austria), 100 U/mL of penicillin and 100 µg/mL of streptomycin (PAA Lab., Austria) and cells were incubated at 37°C with 5% CO_2_ in a humidified atmosphere (Shel Lab., USA).

### Neutral Red Uptake (NRU) Assay

The cytotoxicity of *P. watsonii* extracts, fractions and sub-fractions were measured by Neutral Red Uptake (NRU) assay which was based on the uptake and subsequent lysosomal accumulation of the supravital dye, neutral red in the viable and uninjured cells. The quantification of the dye extracted from the cells has shown to be linear with the cell numbers, both by direct cell count and by protein determinations of cell population [31]. Cells were seeded in a 96-well micro titer plate (Nunc, Denmark) at a concentration of 30,000 cells/mL and placed in a CO_2_ incubator at 37°C to allow the cells to adhere before addition of the the extracts, fractions and sub-fractions of *P. watsonii*. After 3 hours, cells were treated with extracts and fractions of *P. watsonii* at six different concentrations i.e., 1, 10, 25, 50, 75 and 100 µg/mL and incubated for 72 hours. Wells containing untreated cells (without addition of any extract) were regarded as a negative control, whereas cells treated with doxorubicin (0.5–10 µg/mL) served as a positive control. At the end of the incubation period, the medium was replaced with medium containing 50 µg/mL Neutral Red (NR) solution (50 µg/mL NR in culture media, 24 hours pre-incubated in the dark at room temperature and then centrifuge at 1500 g for 10 min before use) and incubated for further 3 hours to allow for uptake of the vital dye into the lysosomes of viable and uninjured cells. The medium was then removed and cells were rapidly washed with the calcium chloride-formaldehyde mixture. The dye within viable cells was eluted from the cells with a mixture of acetic acid, ethanol and water (1∶50:49) (0.2 ml). The plates were agitated on a micro titer plate shaker (LT BioMax 500) for 30 min and then absorbance against a blank reference was measured at 540 nm using a micro plate reader (Emax, Molecular Devices, USA).

NR uptake, proportional to the number of viable cells within the well, was expressed as a percentage of uptake by control cells [(OD_control_ – OD_sample_)/(OD_control_) × 100%]. IC_50_ values (concentration required to reduce cells viability by 50% as compared to the control cells) for each extract was extrapolated from the graphs plotted using the OD values obtained. In order to investigate whether the cytotoxic activity was specific to the cancer cells, the cytotoxic activity of the extracts, fractions and sub-fraction was tested and the selectivity index (SI) of active extract was determined. The selectivity index (SI) of the extracts is defined as the ratio of cytotoxicity (IC_50_ values) on normal lung fibroblast (MRC-5) cells to cancer cells (SKOV-3, Ca Ski and HT-29). (SI = IC_50_ on MRC-5 cells/IC_50_ on cancer cells). Samples with an SI greater than 3 were considered to have high selectivity towards cancer cells [32].

### Detection of Apoptosis

#### Morphological assessment of apoptotic cells by phase-contrast inverted microscope

Cells (3×10^4^ cells/mL) were incubated for 24 hours in the absence or presence of PW-M, PW-H and PW-E of *P. watsonii* and sub-fraction PPWH-7 at concentrations of 10.0 µg/mL in 24-well tissue culture plates. At the end of the incubation period, the media was removed and cells were washed once with a phosphate buffer saline (PBS pH 7.4) and observed under a Leica DMI 3000B phase-contrast inverted microscope (Leica Microsystems, Germany) at 200× magnifications and photographed.

#### Morphological assessment of apoptotic cells by acridine orange (AO)-ethidium bromide (EB) double staining

Cell morphological changes were assessed by differential staining using DNA-intercalate fluorescent dyes, acridine orange (AO) and ethidium bromide (EB). Cells (3×10^6^ cells/mL) were seeded in 24-well tissue culture plates, treated with PW-M, PW-H, and PW-E and PPWH-7 at concentration of 10.0 µg/mL and incubated for 24 hours. Untreated cells were used as a negative control. After incubation, control and treated cells were pelleted and suspended in 25 µl of PBS pH 7.4. To each sample, 1 µl of AO/EB solution (1 part of 100 µg/mL of AO in PBS; 1 part of 100 µg/mL of EB in PBS) was added prior to microscopic examination. The cell suspension was placed on a 3-well Teflon coated microscopic slides, covered with a glass cover slip, and was observed and photographed with a Nikon Eclipse 80i (Nikon, NY) under fluorescence illumination with triple filters (FITC, Cy3 and DAPI). Images were analyzed by Nikon’s Imaging Software, NIS-Elements (Nikon, NY).

### DNA Fragmentation Analysis by Agarose Electrophoresis

Cells were treated in the presence or absence of PW-M, PW-H, PW-E and PPWH-7 (1.0, 10.0 and 100.0 µg/mL) for 48 hours, then harvested by using 500 µl lyses buffer [50 mM Tris-HCL (pH 8.0), 20 mM EDTA (pH 8.0), 1.43 ml of Tergitol^®^ solution Type NP-40, and 20 µl of SDS 10%] at 65°C for 30 min on ice. Subsequent steps were carried out on ice. 100 µl of 8 M potassium acetate was added to the suspended mixtures and incubated on ice for 1 hour. The supernatant was obtained by centrifuging the lysates at 10,000 ×g for 10 min (Heraeus PICO 17, Thermo Scientific, USA) and the soluble proteins were extracted by phenol/chloroform/isoamyl alcohol (PCI; 25∶24:1, v/v/v) (Invitrogen Life Technologies). Finally, DNA was precipitated using 2× volume of ice-cold absolute ethanol. To perform the DNA fragmentation assay, the DNA pellet was dissolved in 20 µl of TE buffer [10 mM Tris-HCl and 1 mM EDTA, pH 8.0] together with 1 µl of RNAse (10 µg/mL) and 1 µl of Proteinase K (100 µg/mL) and incubated at 37°C for 30 min. DNA fragmentation was analyzed by 1.5% agarose gel electrophoresis. DNA bands were observed and photographed using a Gene Flash gel documentation system (Syngene Bioimaging, UK). Apoptosis induction is indicated by the appearance of DNA ladder fragments of approximately 180–200 bp multiples on the agarose gel.

### Caspase-3 Activity Assay

Activity of caspase-3 was determined using the Caspase-3/CPP32 colorimetric assay kit (Biovision, CA) according to manufacturer’s protocol. The assay is based on spectrophotometric detection of the chromophore, *p*-nitroanilide (*p*NA), after its cleavage from the labeled substrate DEVD-*p*NA. Cells (2×10^6^) were pelleted and lysed after treatment with the 10 µg/mL of PW-M, PW-H, and PW-E of *P. watsonii* and sub-fraction PPWH-7 for 48 hours. Assays were performed on 96-well microtiter plates by incubating 100 µg protein of cell lysate per sample in 50 µl of 2× reaction buffer. The reaction buffer is supplemented with 10 mM DTT and substrates of 4 mM DEVD-pNA in a final volume totaling to 100 µl and incubated at 37°C for 1.5 hours. Formation of *p*-nitroanilide was measured using the ELISA micro-plate reader at a wavelength of 405 nm and the caspase activities were expressed as percentage of enzyme activity compared with control (untreated cells).

### Cell Cycle Analysis by Flow Cytometry

To compare the effects of extract PW-H and sub-fraction PPWH-7 on the cell cycle, the CycleTEST™ PLUS DNA Reagent Kit (Becton Dickinson, USA) was used. The cells (1×10^6^ cells/mL) were seeded in a 6-well plate and treated with 10 µg/mL of PW-H and sub-fraction PPWH-7. After 24 hours incubation, the cells were harvested, brought to suspension, permeabilized with trypsin buffer. The nuclear DNA was labeled with propidium iodide (PI) stain solution and incubated in the dark between 2° to 8°C for 10 min. Cell cycle phase distribution of nuclear DNA was determined by flow cytometry (Becton Dickinson FACS Calibur, USA) by analyzing at least 10,000 cells per sample. The percentage of cells in G1, S and G2 phases were analyzed by ModFit LT software (Verity Software House, Topsham, ME).

### Statistical Analyses

Data was presented as mean ± standard deviation. Significant differences were determined by using the Student’s *t*-test where *p < 0.05 denotes a statistically significant difference. All the samples were measured in triplicates.

## Results

### Cytotoxicity of *P. watsonii* Extracts-NRU Assay

In the present study, the potential cytotoxic effects (IC_50_) of *P. watsonii* extract in methanol (PW-M), hexane (PW-H) and ethyl acetate (PW-E) as well as fractions and sub-fractions of PW-H were investigated on two human gynecologic cancer cells (SKOV-3 and Ca Ski), one colon cancer cells (HT-29) and one normal cells (MRC-5) by using the NRU assay. Based on the US National Cancer Institute guidelines, a crude extract is generally considered to have *in vitro* cytotoxic activity if the IC_50_ value in carcinoma cells, following incubation between 48 and 72 hours, is ≤20 µg/mL, while for a pure compound the IC_50_ value is ≤4 µg/mL [33]–[34]. Cytotoxic activity (IC_50_) and selectivity index of the extracts, fractions and sub-fractions are summarized in Table 2. In addition, doxorubicin, a commonly used chemotherapeutic drug for the treatment of acute leukemia, lymphomas and different types of solid tumors such as breast, liver and lung cancers [35], was used as a positive control in the experiments. *P. watsonii* extracts exhibited greatest cytotoxic activity on all cancer cells tested where all showed IC_50_ values <20 µg/mL. The most promising and most selective cytotoxic activities were detected in PW-H and PW-E. The IC_50_ (µg/mL) and SI values with PW-H on SKOV-3, Ca Ski and HT-29 cells were 5.79 ± 0.29 (SI = 9.9), 6.94 ± 0.96 (SI = 8.3) and 11.79 ± 1.61 (SI = 4.9), respectively. Whereas the values with PW-E were 5.52 ± 0.50 (SI = 6.1), 3.58 ± 1.01 (SI = 9.4) and 5.14 ± 0.36 (SI = 6.6), respectively. PW-H was selected for the bioassay-guided fractionation as it showed the strongest cytotoxic effect on cancer cells and was the least toxic on normal cells compared to PW-M and PW-E. These are the criteria normally considered to justify further purification of the extract [36].

**Table 2:** Cytotoxicity (IC_50_)^a^ of the extracts of *P. watsonii* and fractions and sub-fractions of PW-H.

Test agents	Cell lines IC_50_ (µg/mL) (SI^b^)			
	SKOV-3	Ca Ski	HT-29	MRC-5
PW-M^c^	**8.51 ± 0.50 (5.8)**	**8.03 ± 0.87 (6.1)**	**18.33 ± 1.53 (2.7)**	49.33 ± 5.80
PW-H	**5.79 ± 0.29 (9.9)**	**6.94 ± 0.96 (8.3)**	**11.79 ± 1.61 (4.9)**	57.30 ± 2.57
PW-E	**5.52 ± 0.50 (6.1)**	**3.58 ± 1.01 (9.4)**	**5.14 ± 0.36 (6.6)**	33.79 ± 2.57
PWH-1^d^	89.77 ± 1.25	>100	>100	>100
PWH-2	**6.80 ± 0.29**	>100	>100	>100
PWH-3	**5.26 ± 0.76**	85.52 ± 3.04	66.25 ± 2.02	>100
PWH-4	**0.29 ± 0.06 (56.0)**	**13.19 ± 3.25 (1.2)**	**10.78 ± 0.76 (1.5)**	**16.25 ± 1.76**
PWH-5	**0.18 ± 0.06 (68.1)**	**7.20 ± 1.15 (1.7)**	**5.20 ± 0.76 (2.4)**	**12.26 ± 2.52**
PWH-6	**0.42 ± 0.12 (18.7)**	**5.51 ± 0.50 (1.4)**	**5.68 ± 0.76 (1.4)**	**7.84 ± 1.04**
PWH-7	**0.77 ± 0.29 (10.4)**	**8.78 ± 0.58 (0.9)**	**1.21 ± 1.04 (6.6)**	**8.02 ± 1.80**
PWH-8	**0.36 ± 0.12 (23.0)**	**2.77 ± 0.76 (3.0)**	**1.19 ± 0.29 (6.9)**	**8.27 ± 1.04**
PWH-9	69.50**±**1.72	>100	>100	>100
PWH-10	>100	>100	>100	>100
PPWH-1^e^	100.00 ± 2.89	>100	72.81 ± 6.66	>100
PPWH-2	>100	>100	>100	>100
PPWH-3	74.01 ± 8.53	98.84 ± 2.02	>100	>100
PPWH-4	37.03 ± 0.50	60.02 ± 2.60	40.82 ± 2.08	>100
PPWH-5	**4.75 ± 2.57**	45.18 ± 3.18	63.78 ± 2.08	>100
PPWH-6	**9.52 ± 1.80 (1.5)**	**12.04 ± 3.50 (12.1)**	**14.76 ± 4.19 (1.0)**	**14.54 ± 1.80**
PPWH-7	**0.66 ± 0.06 (15.5)**	**0.83 ± 0.00 (12.3)**	**0.83 ± 0.10 (12.3)**	**10.21 ± 1.26**
PPWH-8	**0.88 ± 0.10 (19.1)**	**9.16 ± 0.58 (1.8)**	**18.31 ± 2.75 (0.9)**	**16.83 ± 1.26**
Doxorubicin	**0.42 ± 0.24 (4.1)**	**0.68 ± 0.08 (2.5)**	**0.63 ± 0.03 (2.7)**	**1.72 ± 0.08**

a Data are presented as mean ± SD from 3 independent experiments, triplicate for each. Values in bold characters are considered to have cytotoxic activity (IC_50_ ≤ 20 µg/mL).

b Selectivity index.

c PW-M, PW-H & PW-E: extracts of *P. watsonii* in 3 different solvents, MeOH, hexane and EtOAc, respectively.

d PWH-1, PWH-2,…….,PWH-10: fractions of PW-H of *P. watsonii.*

e PPWH-1, PPWH-2,….,PPWH-8: sub-fractions from pooled fractions (PWH-4,…,PWH-8).

### Bioassay-guided Fractionation-NRU Assay

Column chromatography (CC) of the PW-H yielded a total of 10 fractions. Fractions of PW-H demonstrated stronger cytotoxic activity and higher selectivity when compared to the PW-H particularly when tested on SKOV-3 cells (Table 2). On SKOV-3 cells, the fractions PWH-4, PWH-5, PWH-6, PWH-7 and PWH-8 exhibited IC_50_ values of 0.29 ± 0.06 (SI = 56.0), 0.18 ± 0.06 (SI = 68.1), 0.42 ± 0.12 (SI = 18.7), 0.77 ± 0.29 (SI = 10.4) and 0.36 ± 0.12 (SI = 23.0) µg/mL, respectively. IC_50_ values obtained when Ca Ski and HT-29 cells were treated with the fractions PWH-4 – PWH-8 were between the range of 2.77–13.19 and 1.21–10.78 µg/mL, respectively. The fractions PWH-4 to PWH-8 were obtained from the mid portion of the silica column (hexane/ Me_2_CO: 40∶60/50∶50) and this shows that the presence of highly cytotoxic compounds were at the intermediate polarity phase. These fractions were pooled and subjected to the second step of bioassay-guided fractionation and yielded 8 sub-fractions. PPWH-7 was the most cytotoxic and exhibited higher selectivity (SI) on Ca Ski and HT-29 cells with IC_50_ (µg/mL) and SI values of 0.83 ± 0.00 (SI = 12.8) and 0.83 ± 0.10 (SI = 12.8), respectively (Table 2). On the contrary, purified sub-fraction PPWH-7 demonstrated lower cytotoxic and selectivity effect on SKOV-3 cells (IC_50_ = 0.66 ± 0.06 µg/mL; SI = 15.5) compared to the fractions PWH-4 (IC_50_ = 0.29 ± 0.06 µg/mL; SI = 56.0), PWH-5 (IC_50_ = 0.18 ± 0.06 µg/mL; SI = 68.1), PWH-6 (IC_50_ = 0.42 ± 0.12 µg/mL; SI = 18.7) and PWH-8 (IC_50_ = 0.36 ± 0.12 µg/mL; SI = 23.0). It is worth mentioning that PPWH-7 showed similar cytotoxic activity as the standard anticancer drug, doxorubicin which was used as a positive control in the present study. However doxorubicin also exhibited high toxicity on MRC-5 normal cells whereas PPWH-7 showed about 6 times lower toxicity on the MRC-5 normal cells.

### LCMS/MS Analysis

**Figure 3:**
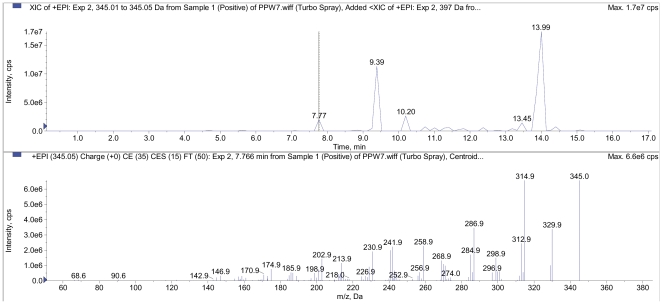
An example of the LC-MS/MS analysis showing a positive full spectra chromatogram of ellagic acid detected in EPI mode.

**Figure 4:**
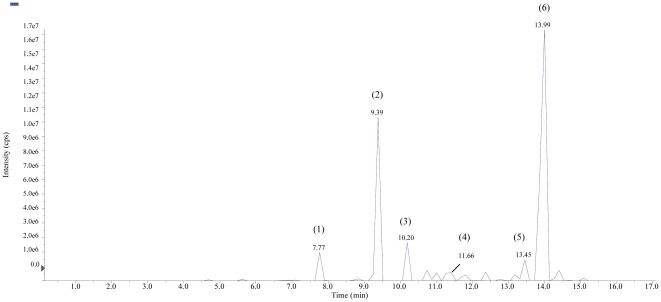
LC-MS/MS MRM chromatogram of sub-fraction PPWH-7. Peak numbers refer to Table 3.

Sub-fraction PPWH-7 was analyzed by LC-MS/MS system and at least 15 compounds were present in PPWH-7 of which 7 compounds with retention times of major peaks were identified through mass spectrometry (Table 3). An example of the LC-MS/MS analysis showing a positive full spectra chromatogram of one of the detected compound (ellagic acid in sub-fraction PPWH-7) in a positive ion mode (EPI mode) is shown in Figure 3. The major peak with a retention time of 13.99 min was identified as methyl ester of geraiinic acid (MS *m/z*  =  397), and potassium phyllanthin (MS *m/z*  =  457). Phyllanthin was also identified as sodium phyllanthin (MS *m/z*  =  441) at a retention time of 11.66 min. Another major peak was identified as sterol glucoside (MS *m/z*  =  718), an isoprenoids at retention time of 9.39 min. Two triterpene were eluted at retention time of 10.20 min and 13.45 min and were identified as glochidone (MS *m/z*  =  423) and betulin (MS *m/z*  =  443), respectively. A polyphenolic compound identified as trimethyl ether of ellagic acid (MS *m/z*  =  345) was eluted at retention time of 7.77 min (Figure 4).

**Table 3:** Identification of compounds in PPWH-7 by using LC-MS/MS data.

Peak	Retention time(min)	MW(*m/z*)	MS/MS	Tentative ID*
1	7.77	345	330, 315	trimethyl ether of ellagic acid
2	9.39	718	701, 641, 613, 581	sterol glucoside
3	10.20	423	405, 381, 363	glochidone
4	11.66	441	423, 405, 395, 339, 315	sodium phyllanthin
5	13.45	443	425, 369	betulin
6	13.99	397	379, 353, 339, 327	methyl ester of geraiinic acid
6		457	439, 397, 379, 369, 353	Potassium phyllanthin

* Identification were aided by comparison with reference standards where available and by correlation with previous literature reports [24]–[30].

### Morphological Assessment of Apoptotic Cells by Phase-contrast Inverted Microscope

**Figure 5:**
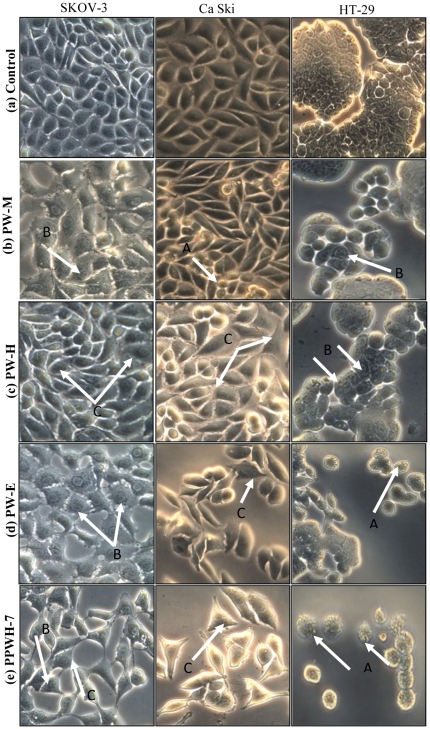
Morphological observation by inverted phase-contrast microscope (200×). SKOV-3, Ca Ski and HT-29 cells after treatment with 10 µg/mL of PW-M, PW-H, PW-E and PPWH-7 for 24 hours. Arrows indicate the formation of (A) apoptotic bodies; (B) condensed nuclei and (c) membrane blebbing as evidence of apoptosis. Images are representative of one of three similar experiments.

Morphological observation of untreated SKOV-3, Ca Ski and HT-29 cells showed that the control cells maintained their original morphology which are cuboids and polygonal in their shape and were adherent to the plates (Figure 5). In contrast, SKOV-3, Ca Ski and HT-29 cells-treated with PW-M, PW-H, and PW-E and PPWH-7 exhibited apoptotic-like characteristics such as shrinkage of cells, bleb formation and nuclear chromatin condensation and aggregation into dense masses beneath the nuclear membrane. Cells undergoing apoptosis also resulted in other types of morphological changes such as shrinkage of rounded up cells and losing contact with neighboring cells. As a result, some sensitive cells even detached from the surface of the well plates.

### Morphological Assessment of Apoptotic Cells by Acridine Orange (AO)-ethidium Bromide (EB) Double Staining

**Figure 6:**
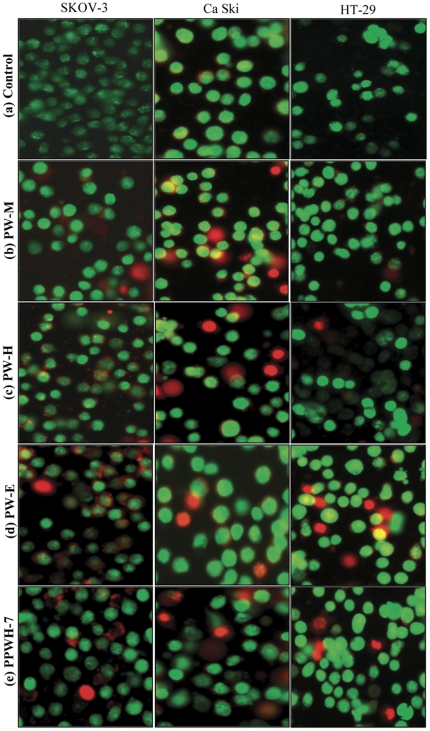
Morphological observation with AO/EB double staining by fluorescence microscope (200×). Control cells and cells treated with PW-M, PW-H, PW-E and PPWH-7 at 10.0 µg/mL for 24 hours. Live cells with uniform bright green nuclei and cytoplasm were observed in control group (a). Apoptotic cells with condensed chromatin are uniformly fluorescent whereas fragmented nuclei and necrotic cells stained bright orange were observed in cells treated with the test agents studied (b–e). Images are representative of one of three similar experiments.

The morphological changes of the SKOV-3, Ca Ski and HT-29 cells treated with 10.0 µg/mL of PW-M, PW-H, PW-E and PPWH-7 for 24 hours were observed by AO/EB staining and the cells were classified as live (normal), apoptotic and necrotic (Figure 6). Live cells with intact DNA and nucleus will have a round and green nuclei. Apoptotic cells will have condensed chromatin which gives several green colored nuclei and the DNA of the necrotic cells would be stained bright orange. As shown in Figure 4, the nucleus of the control cells was smoother and uniformly bright green together with the cytoplasm. Cells treated with extracts and sub-fraction exhibited characteristic changes and showed signs of apoptosis, i.e., cell shrinkage, nuclear condensation and fragmentation. The nucleus of the treated cells shown as bright green with highly condensed chromatin is uniformly fluorescent. The condensed chromatin can be observed in the form of crescents around the periphery of the nucleus or the entire chromatin is present as one or a group of featureless, bright spherical beads. The number of cells stained bright orange were generally very low in all the treatments which indicated that most of cells were not undergoing necrosis but cell death occurred primarily through apoptosis.

### DNA Fragmentation Analysis by Agarose Electrophoresis

**Figure 7:**
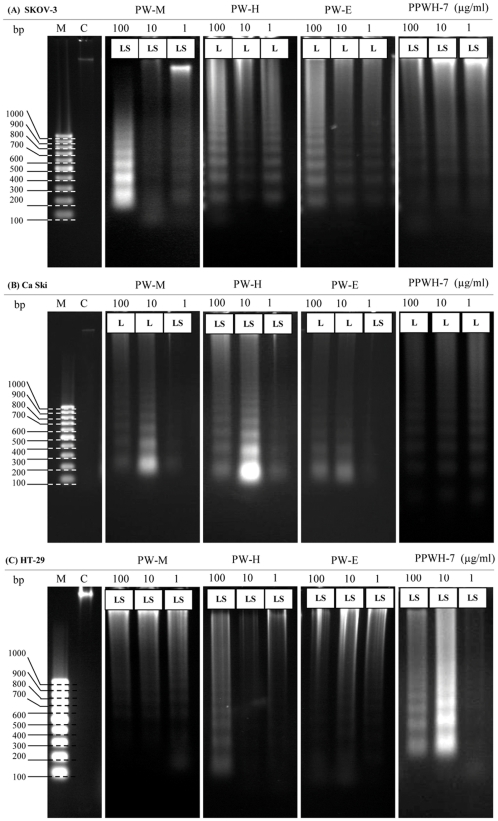
Effects of PW-M, PW-H, PW-E and PPWH-7 on DNA fragmentation on cells. Cells were treated with indicated concentrations of the test agents for 48 hours and genomic DNA was extracted and separated using 1.5% agarose gel electrophoresis. L = DNA ladder; LS = diffused DNA ladder with interspersed smearing; M = 100 bp DNA marker and C = untreated control cells.

An important feature of cell apoptosis is the fragmentation of genomic DNA into integer multiples of 180–200 bp units producing a characteristic ladder on agarose gel electrophoresis [37]. To elucidate whether PW-M, PW-H and PW-E and PPWH-7 decrease cell survival by the induction of DNA fragmentation, genomic DNA isolated from SKOV-3, Ca Ski and HT-29 cells were exposed to different concentrations of extracts, electrophoresed and photographed (Figure 7). Typical DNA ladder formation, a hallmark of apoptosis, can be seen clearly in the SKOV-3 cells treated with PW-H and PW-E and in the Ca Ski cells treated with PW-M, PW-E and PPWH-7. The DNA ladder was observed less clearly in all the treated HT-29 cells as there were interspersing smear in the lanes. The smearing could be due to some post-apoptotic cell necrosis. In comparison, the DNA from untreated cells did not exhibit any fragmentation or smearing.

### Activation of Caspase-3

**Figure 8:**
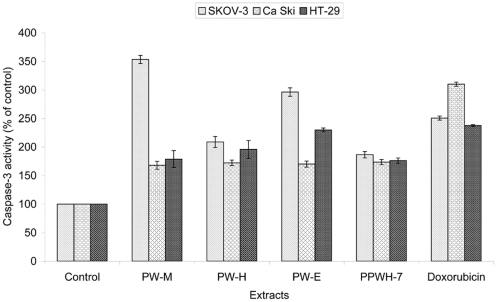
Caspase-3 activity in cancer cells was analyzed using Caspase-3/CPP32 Colorimetric Assay Kit. Multiple increases in caspase-3 activity in cells after treatment with 10 µg/mL of extracts for 48 hours and was determined by comparing the results with control. The values are expressed as means ± SD in three independent experiments. Significant differences were compared between control and treated cells (*p<0.05).

To ascertain whether the cytotoxic activity could be dependent by the activation of caspase-3 which plays a central role in mediating apoptotic responses [38], we measured the intracellular levels of caspase-3 in SKOV-3, Ca Ski and HT-29 cells after being induced with studied extracts. Following 48 hours treatment of SKOV-3, Ca Ski and HT-29 cells with various concentrations of PW-M, PW-H, PW-E and PPWH-7, caspase-3 activities were measured and compared with control cells (Figure 8). As shown, SKOV-3 cells treated with PW-M, PW-H, PW-E and PPWH-7 exhibited a dramatic increment in caspase-3 activity ranging from 1.9 to 3.5-folds higher compared to untreated cells. The activity in PW-M and PW-E was 3.5 and 2.9-folds higher than untreated cells, respectively. These values are even higher than the doxorubicin-treated cells which had only 2.5-folds increase in activity. The caspase-3 activity increased slightly (1.7-folds) in all Ca Ski cells treated with PW-M, PW-H, PW-E and PPWH-7. In HT-29 cells, treatment with PW-E caused the highest increment in caspase-3 activity (2.3-folds) followed by PW-H (2.0-folds) and PW-M and PPWH-7 (1.8-folds).

### Effects on Cell Cycle Regulation

**Figure 9:**
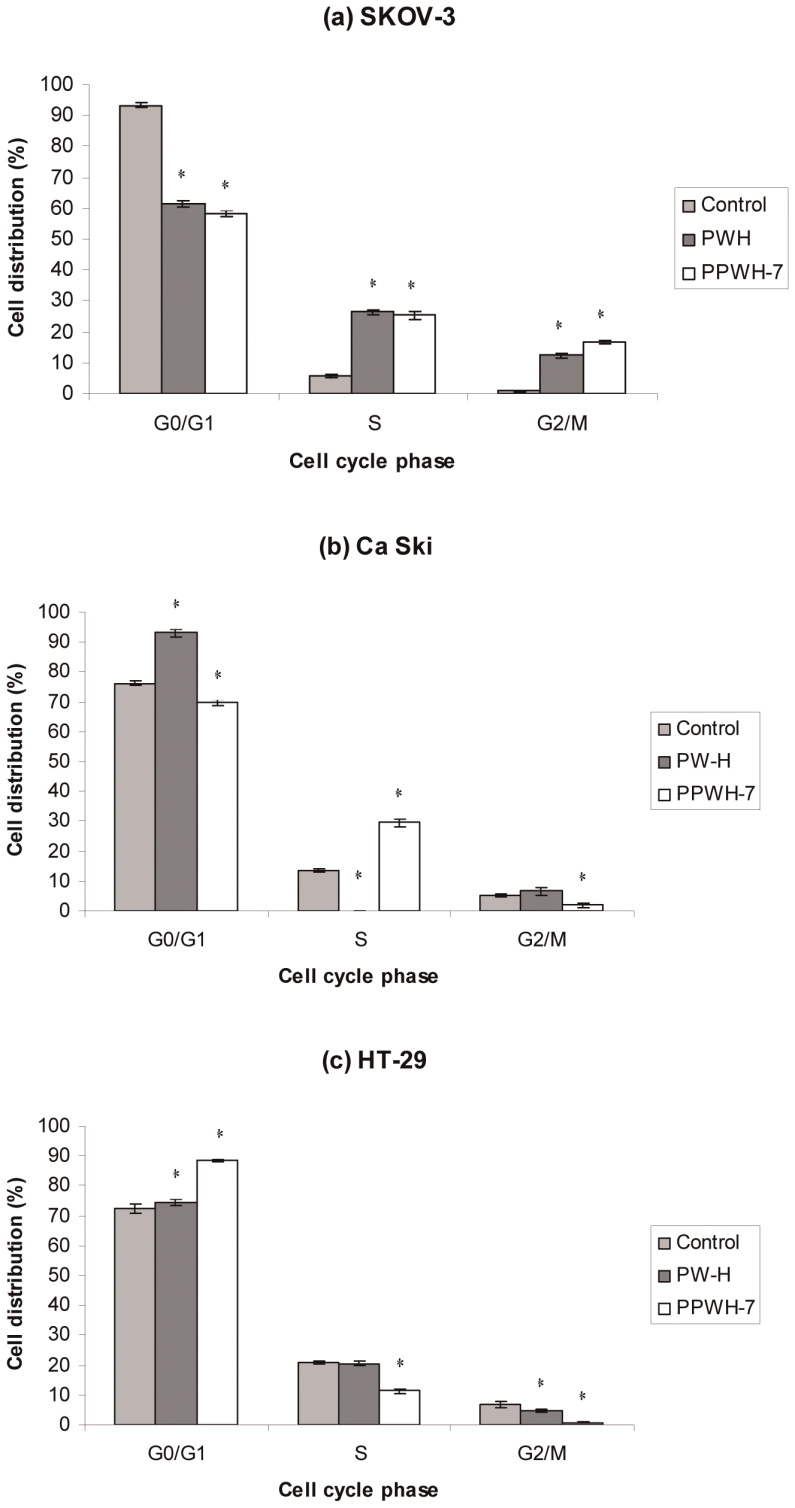
Effects of *P. watsonii* on the cell cycle of (a) SKOV-3, (b) Ca Ski and (c) HT-29 cells. Cells (1×10^6^ cells/mL) were incubated in the absence (control) or presence of PW-H and PPWH-7 for 24 hours and then analyzed by flow cytometry. Data are representative of one of the three similar experiments. Significant differences were compared between control and treated cells (*p<0.05).

Since PW-H and sub-fraction PPWH-7 exhibited the most promising cytotoxicity and shown to be quite selective for cancer cells (Table 2), we next analyzed the regulation of cell cycle distribution in the presence of PW-H and PPWH-7 in SKOV-3, Ca Ski and HT-29 cells by flow cytometry. Interestingly, SKOV-3 cells exhibited a dramatic accumulation of cells in S and G_2_/M phases and a concomitant decrease of the percentage of cells in G_0_/G_1_ phase, suggesting that both PW-H and PPWH-7 induced SKOV-3 cell cycle arrest at S and G_2_/M phases (Figure 9). Significant increment from 5.5 ± 0.50% (control) to 26.4 ± 0.87% and 0.8 ± 0.20% (control) to 12.3 ± 0.64% were observed in S and G_2_/M phases (*p*<0.05), respectively in PW-H-treated cells. Whereas, significant increment from 5.5 ± 0.50% (control) to 25.3 ± 1.16% (*p*<0.05) in S phase and 0.8 ± 0.20% (control) to 16.5 ± 0.60% (*p*<0.05) in G_2_/M phase were also been observed in PPWH-7-treated cells. In the PW-H-treated Ca Ski cells, the number of cells in G_0_/G_1_ phase was 93.0 ± 1.11% compared to control cells (76.3 ± 0.81%) (*p*<0.05). Whereas treatment with PPWH-7 significantly increased the proportions of S phase cells from 13.6 ± 0.60% (control) to 29.6 ± 1.27% (*p*<0.05). These results provided evidence that the observed apoptotic cell death was partly due to the cell cycle arrest in G_0_/G_1_ and S phase induced by PW-H and PPWH-7, respectively. PW-H and PPWH-7 significantly increased the percentage of HT-29 cells in G_0_/G_1_ phase (74.5 ± 1.26% and 88.5 ± 0.57%) (*p*<0.05), respectively, as compared with the control cells (72.6 ± 1.60%), indicating that PW-H and PPWH-7-induced HT-29 cell cycle arrest in G_0_/G_1_ phase.

## Discussion

In the current work, extracts of *P. watsonii* in different solvents from non-polar to polar (MeOH, hexane and EtOAc) demonstrated strong cytotoxicity and high sensitivity towards both of the human gynecologic and colon cancer cells as compared to the normal lung fibroblast cells. These conclusions are drawn based on the lower IC_50_ values (≤20.0 µg/mL) and higher SI values (>3) that were obtained. These results suggest that compounds from *P. watsonii* may serve as a promising new experimental anti cancer agent for the treatment of human cancers. However, as our current studies were focused on *in vitro* investigation of extracts/fractions effects, further research might include exploration of its effects in animal tumor models to confirm its anti cancer activity *in vivo*.

Bioassay-guided fractionation approach which has been recognized as an important method in the attempt to isolate bioactive components from natural sources [39] was used to isolate the most effective constituents from *P. watsonii* that were capable in inhibiting the growth of the human gynecologic and colon cancer cells. The bioassay-guided fractionation of PW-H revealed that cytotoxic compounds were found mainly in the middle polarity phase of the solvent system used (n-hexane/acetone phase). It can be concluded that the non-polar compounds of *P. watsonii* potently inhibited the *in vitro* cell proliferation of SKOV-3, Ca Ski and HT-29. PPWH-7 was the most cytotoxically active sub-fraction and showed similar cytotoxic activity as the standard anti cancer drug, doxorubicin which was used as a positive control in the present study. US National Cancer Institute assigns that a pure compound possessed a significant cytotoxic activity if the IC_50_ value is ≤4 µg/mL [33]–[34]. The IC_50_ values established for PPWH-7 ranged from 0.66–0.83 µg/mL, a highly cytotoxic activity when considering a pure compound and these findings strongly suggest the promising potential of *P. watsonii* to be developed as an anti cancer drug. Variations in the cytotoxic activities among the fractions and sub-fractions against the cancer cells studied were also observed and this could be attributed to the distribution of the several identified cytotoxic compounds in different fractions/sub-fractions. In the present study, it also been clearly observed that fraction PWH-4, PWH-5, PWH-6 and PWH-8 exhibited a higher degree of cytotoxic acivity than purified sub-fraction PPWH-7 towards SKOV-3 cells. While sub-fraction PPWH-7 demonstrated an increased cytotoxic activity and higher selectivity towards Ca Ski and HT-29 cells, the reason for the decreased cytotoxic activity towards SKOV-3 cells remains to be determined. Synergism between the components and/or the presence of other minor active compound(s) is more likely to have caused the observed cytotoxicity of the fractions and sub-fraction. Further experiments are required to confirm this hypothesis with the cytotoxic activity of the fractions and sub-fraction of *P. watsonii* towards SKOV-3 cells.

At present, LC-MS is one of the most sensitive analytical methods to yield information about the molecular weight as well as the structure of the analyst [40]. Analysis of the PPWH-7 using LC-MS/MS indicated the presence of trimethyl ether of ellagic acid, methyl ester of geraiinic acid, glochidone, betulin, Phyllanthin sodium salt, Phyllanthin potassium salt and sterol glucoside and some of these detected compounds have been shown to have anti cancer activities. Based on this information, we conjecture that the observed cytotoxic activity in *P. watsonii* may be due to the presence of some of these compounds. Plant-derived ellagic acid has previously been identified as a potent anti cancer agent [41]–[44] with suggested molecular targets for ellagic acid effects being NF-κB, cyclin D1, p21^cip1/waf1^ and p53 [45]. Many studies have shown that ellagic acid possessed growth-inhibiting and apoptosis promoting activities against various human cancer cell lines representing different tissues such as Ca Ski cervical carcinoma cells [46], Caco-2 colon cells, MCF-7 breast cells and Hs 578T breast cells, DU 145 prostatic cells [47] and SH-SY5Y neuroblastoma cells [48]. Anti cancer potential of geraiinic acid, a compound under the chemical class of monoterpene [49] has not been reported but there are numerous studies on the cytotoxicity of monoterpenes and its derivatives [50]–[54]. Anti tumor activity of betulin, also known as betulinol, betuline and betulinic acid and its derivatives could be therapeutically important, because these compounds exhibit high cytotoxic activity against several cancer cells. Betulin was found to be cytotoxic against neuroblastoma cell lines [55], triggered apoptotic cascade in human malignant glioma cells [56] and inhibited the growth of neoplastic cell lines, such as ovarian carcinoma (A2780, OVCAR-5 and IGROV-1), lung carcinoma (H460 and POGB) and cervix carcinoma (A431) [57]. Phyllanthin, a lignan compound, is a known principal constituent of *P. niruri* and there have been no data reported on its cytotoxic effect. Sterol glucoside was also been detected in PPWH-7 and sterols isolated from various marine organisms and alga was reported to possess cytotoxic activity [58]–[60].

Many researchers recognized induction of apoptosis, a programmed cell death in cancer cells or malignant tissues, as one of the efficient strategies in cancer chemotherapy and a very important property of a candidate anti cancer drug [11]. In order to evaluate whether the cytotoxic effects of PW-M, PW-H, PW-E and PPWH-7 upon SKOV-3, Ca Ski and HT-29 cells were related with the apoptotic processes, we investigated the effects of these test agents on the induction of apoptotic cell death by various morphological and biochemical methods. All our study indicated that *P. watsonii* inhibited the growth of the human gynecologic and colon cancer cells through apoptosis induction.

Apoptotic cells were first recognized from the characteristic changes in their morphology, particularly changes in the nucleus. Distinct apoptotic morphological changes in treated-SKOV-3, Ca Ski and HT-29 cells include the rounding up of cells, membrane blebbing, chromatin condensation and formation of apoptotic bodies. These observations were made under phase-contrast inverted microscope and with AO/EB double staining by fluorescence microscope. Formation of DNA fragmentation ladder which correlates with the early morphological signs of apoptosis [61] has been widely used as a distinctive marker of the apoptosis process [27]. The typical ladder-like appearance of DNA in the tested cell lines observed in our study reconfirmed the apoptosis-inducing capability of both the extracts and sub-fraction. The induction of apoptosis is almost always associated with the activation of caspases; the cytoplasmic aspartate-specific cysteine protease [62]–[63]. The release of cytochrome *c* from mitochondria to cytosol after being induced by a variety of apoptosis-inducing agents leads to the formation of apoptosome composed of cytochrome *c*, apoptosis protease activating factor-1 (Apaf-1), deoxyadenosine triphosphate (dATP), and procaspase-9, which forms a platform for the efficient processing and activation of caspase-9 [64]–[66]. Activation of caspase-9, in turn, cleaves effectors caspases such as caspase-3, -6, and -7 [66]. Caspase-3 activates other caspases, cleaves cytoskeletal proteins, or activates the caspase-activated DNase. In particular, caspase-3 cleaves an inhibitor of caspase-activated DNase that allows caspase-activated DNase to enter the nucleus and to fragment nuclear DNA and culminate in the orderly demise of the cell [67]. The present study indicated that following the addition of extract of *P. watsonii* sub-fraction PPWH-7, SKOV-3, Ca Ski and HT-29 cells displayed an increment in the activity of caspase-3. Many plants as well as other *Phyllanthus* species extracts have shown to induce apoptosis in various cell types by inducing the activity of caspase-3 protein [68]–[70] and this is the first report on *P. watsonii*.

Cell cycle is an essential process in the development, differentiation, and proliferation of mammalian cells [71]. Since deregulation of the cell cycle machinery has been associated with cancer initiation and progression [72], suppression of the cell cycle has become an appreciated target for management and treatment of tumor cells with cytotoxic agents [71]. It has been reported that many anti cancer agents arrest cell cycle at the G_0_/G_1_, S or G_2_/M phases and then induce apoptotic cell death [73]–[76]. Our data suggest that PW-H and PPWH-7 induced cell cycle arrest at S-G_2_/M phase in SKOV-3 cells and at G_0_/G_1_ phase in HT-29 cells. Whereas in Ca Ski cells, PW-H induced cell cycle arrest at G_0_/G_1_-G_2_/M phases and PPWH-7 at S phase. Cell cycle progression is regulated by the activity of cyclins, a family of proteins that activate the cell-cycle-dependent kinases (Cdks) [77]. Cyclin A is required for the S phase and passage through G_2_ phase and cyclin E activates cdk2 protein near the start of the S phase [78]. Cyclin D1 is known to bind and activate cdk4, which is largely involved in controlling the G_1_/S restricting point whereas the G_2_/M transition is positively regulated by the cdk2 and cyclin B complex [79]. Cdk-cyclin complexes are negatively controlled by the Kip/Cip family of cyclin-dependent kinase inhibitors (CDKIs), namely p27^Kip1^ and p21^Cip1^ [80]. In order to elucidate the mechanism of action of extract on cell cycle and the changes of multiple regulatory molecules in the cell cycle, further work should be carried out.

In conclusion, this study clearly demonstrates for the first time that the sub-fraction of *P. watsonii* strongly inhibits the growth and induces apoptosis in human gynecologic and colon cancer cells. Additional studies are warranted to understand the interaction mechanism involved in the apoptotic signaling pathway induced by *P. watsonii*. Further studies on the isolation of pure compound(s) and chemical characterization of the cytotoxically active compounds of *P. watsonii* are currently undergoing.
